# Rapid Qualitative Analysis to Inform Preimplementation of a Stepped‐Care Model for Depression Care Among Older People Living With HIV in Ibadan, Nigeria

**DOI:** 10.1155/arat/9573277

**Published:** 2026-10-07

**Authors:** Olufisayo Oluyinka Elugbadebo, Faithful Moposi Akintade, Lauren B. Beach, Bibilola D. Oladeji, Lisa M. Kuhns

**Affiliations:** ^1^ Department of Psychiatry College of Medicine University of Ibadan Ibadan Nigeria ui.edu.ng; ^2^ Institute of Child Health College of Medicine University of Ibadan Ibadan Oyo Nigeria ui.edu.ng; ^3^ Department of Medicine Social Sciences and Department of Preventive Medicine Northwestern University Feinberg School of Medicine Chicago Illinois USA northwestern.edu; ^4^ Department of Pediatrics Division of Adolescent and Young Adult Medicine Feinberg School of Medicine & Ann & Robert H. Lurie Children’s Hospital of Chicago Northwestern University Chicago Illinois 60611 USA northwestern.edu

**Keywords:** HIV, implementation research, Nigeria, older adults, rapid qualitative analysis, stepped-care model, task-sharing

## Abstract

**Background:**

Depression is common among older adults living with HIV (OLHIV) and is associated with poor antiretroviral adherence and higher viral load. Despite the importance of addressing psychological health issues in older people living with HIV, there is a paucity of tailored depression interventions for low‐resource settings. Although the stepped‐care task‐sharing (SCT) intervention model is effective for depression in low‐resource settings, it has not been applied to this population. This study examined preimplementation requirements for piloting a SCT model for depression among OLHIV in a government‐funded clinic in Southwest Nigeria.

**Methods:**

We conducted a qualitative preimplementation study, guided by the Consolidated Framework for Implementation Research (CFIR), to assess the contextual fit and feasibility of integrating a SCT intervention for depression into routine HIV services. In‐depth interviews were conducted with healthcare providers and older adults with HIV at the Infectious Disease Institute, Ibadan, Nigeria. Data were analyzed using rapid qualitative methods to identify CFIR‐informed barriers, facilitators, and adaptation needs to guide intervention refinement prior to pilot testing.

**Results:**

Twenty‐two interviews were conducted with healthcare providers (*n* = 11) and OLHIV (*n* = 11), each lasting 30–45 min. Findings indicated that the SCT intervention was perceived as feasible, supported by staff willingness and existing clinic structures that could be leveraged during pilot testing. Key barriers to implementation included high workload, limited provider training, stigma, discontinuity of care, and financial constraints. Facilitators included organizational readiness and high acceptability among service users.

**Conclusion:**

The SCT model is perceived as acceptable and potentially feasible for integrating depression care into HIV services for OLHIV. However, it requires adaptation into existing workflows, provider capacity, and patient follow‐up routines. Findings provide insights to refine the intervention processes in preparation for pilot testing and integration of psychological healthcare into HIV services.

## 1. Introduction

Sub‐Saharan Africa bears the highest burden of HIV globally, and the population of older adults living with HIV (OLHIV) in the region is projected to increase by nearly 50% by 2030 [1]. As people age with HIV, they face multiple comorbidities, including psychological health conditions such as depression.

Depression is common among people living with HIV (PLWH) [2], and its management is compounded by challenges such as pill burden, missed appointments [3], morbidities [4], economic burden [5], and stigma [5]. Due to these challenges, PLWH often encounter issues related to psychological health symptoms [6]. In sub‐Saharan Africa, including Nigeria, mental health services within HIV care have largely focused on pregnant women, adolescents, young adults, and special population with few services specifically targeting OLHIV. Consequently, their mental health needs often remain unmet. Since the inception of HIV/AIDS Care in Nigeria, HIV care in Nigeria is delivered through a decentralized network of antiretroviral therapy (ART) clinics located in tertiary hospitals, secondary facilities, and selected primary health centers. Since adopting the differentiated service delivery (DSD) policy [7] in 2019, Nigeria has implemented facility and community‐based DSD models that tailor HIV care to the needs of PLWH while reducing health system burden [8]. HIV primary care has improved over the years through interventions such as periodic viral load testing, CD4 quantification, morbidity testing, enhanced adherence counseling, and treatment support [9], driven by widespread training and implementation of the Nigerian HIV care guidelines [10]. Despite these advances, scalable and supportive psychological health interventions remain largely unimplemented [11]. Stepped‐care and task‐sharing (SCT) is a staged approach to delivering care that tailors treatment to symptom severity and patient’s response [12]. It involves providing different levels of treatment intensity, starting with less intensive treatment and stepping up to more intensive treatments only when clinically needed. This model also incorporates task sharing, whereby nonspecialist frontline healthcare providers (HCPs) deliver structured psychosocial interventions [13], while the few available specialists provide training, support, and supervision and handle very severe cases with the most intensive treatment. This model has been proven feasible and effective in resource‐constrained settings and offers a potential solution, having demonstrated effectiveness in managing depression in LMICs [14].

Although effective in primary care settings, these approaches have not been widely applied among older adults or in HIV clinic settings, highlighting a critical gap in psychological health service delivery for this growing older population. The use of this model in OLHIV requires careful contextual adaptation [12], as successful implementation depends on proper planning and tailored strategies [15]. Intervention design must consider patient needs [16], existing care pathways, and potential barriers and facilitators [17]. For this study, a preimplementation needs assessment was conducted to guide the integration of the SCT into existing HIV services before pilot testing its implementation. Rather than assuming that the SCT model would readily fit into an HIV clinical setting, this approach prioritizes stakeholder participation, contextual assessment, and protocol refinement before implementation of this evidence‐based method in Ibadan, Nigeria, ensuring appropriate adaptation prior to pilot testing. This study reports a preimplementation assessment to determine the feasibility and contextual fit of an existing SCT evidence‐based model for treatment of depression within routine HIV primary care by drawing on perspectives of HCPs and OLHIV in a low‐resource setting; the aim of the study is to identify necessary contextual adaptations to inform pilot testing.

## 2. Materials and Methods

This study is a preparatory phase of a pilot study to implement a depression care model into existing HIV services. We conducted qualitative interviews guided by the Consolidated Criteria for Reporting Qualitative Research (COREQ) [18] guidelines and checklist [19] to ensure comprehensive and transparent reporting.

### 2.1. Study Setting

All project activities were conducted at the HIV clinic of the Infectious Disease Institute (IDI), Ibadan, Nigeria. IDI is a major referral center for HIV care serving approximately 6540 PLWH as of June 2026. Approximately 53% of the clinic population comprises adults aged 50 years and older. The clinic provides HIV services, including clinical care, counseling, laboratory diagnostics, pharmacy, and follow‐up through a multidisciplinary team of physicians, nurses, pharmacists, counselors, and records officers and laboratory scientists.

### 2.2. Proposed Intervention

SCT is a tiered approach to delivering care that tailors care to symptom severity and patient’s response [14]. In this model, the intervention is a three‐step care pathway in which treatment intensity is determined by depression severity (PHQ‐9) and response to treatment. Less intensive service interventions are provided first. Participants progress through successive steps according to predefined PHQ‐9 thresholds, with increasing treatment intensity including psychosocial intervention, psychiatric review, antidepressant treatment, and referral where indicated (Table 1).

**TABLE 1:** Proposed intervention.

Step	PHQ‐9 score	Intervention	Reassessment	Next action
Step 1 (baseline)	PHQ‐9 = 10–14	Psychosocial intervention (PI) only for 6 weeks.	6‐week assessment	i. If PHQ‐9 ≥ 10, proceed to Step 2 ii. If PHQ‐9 < 10, continue biweekly PI and complete follow‐up assessments at 3, 6, 9, and 12 months.
	PHQ‐9 ≥ 15	Supervising psychiatrist review + antidepressant (sertraline) + PI for 6 weeks	6‐week assessment	i. If PHQ‐9 ≥ 10, proceed to Step 2 ii. If PHQ‐9 < 10, continue biweekly PI and complete follow‐up assessments at 3, 6, 9, and 12 months.

Step 2	PHQ‐9 ≥ 10 (no previous antidepressant use)	Supervising psychiatrist review + antidepressant + weekly PI for 6 weeks	3‐month assessment	i. If PHQ‐9 ≥ 10, proceed to Step 3. ii. If PHQ‐9 < 10, continue biweekly follow‐up and complete assessments at 6, 9, and 12 months.
	PHQ‐9 ≥ 10 (previous antidepressant use)	Supervising psychiatrist reviews current medication + weekly PI for 6 weeks.	3‐month assessment	i. If PHQ‐9 ≥ 10, proceed to Step 3. ii. If PHQ‐9 < 10, continue biweekly follow‐up and complete assessments at 6, 9, and 12 months.

Step 3	PHQ‐9 ≥ 10	Supervising psychiatrists review every 6 weeks + antidepressant + two PI sessions per week.	Reassessments at 3, 6, 9, and 12 months	Participants with no improvement, refer to a specialist.

### 2.3. Recruitment and Informed Consent

Purposive sampling was used to select HCPs (clinicians, nurses, counselors, and record officers) directly involved in leading or providing care to HIV patients, ensuring participants had relevant experience and insight. Sampled HCPs held permanent positions within the facility and had a minimum of 5 years of experience providing HIV care. For interviews with patients, the nurses assisted in identifying eligible OLHIV, defined as aged 50 years and older who had been receiving HIV care for at least 5 years since diagnosis. Restricting eligibility to patients diagnosed for at least 5 years ensured participants had consistent engagement with HIV services and sufficient clinical experience to reflect on long‐term treatment, comorbidities, and psychosocial needs, while minimizing the potential confounding effects of a recent diagnosis. To ensure HCPs had extensive knowledge of clinic operations and service delivery, we purposively recruited experienced staff, including several who had been working at the HIV clinic. OLHIV with severe cognitive impairment that limited their ability to provide informed consent and participate in the interview were excluded. Prior to the interview, participants were provided with information about the study and provided written informed consent. In addition, verbal informed consent was audio‐recorded just before the commencement of each interview on the scheduled date of interview. Participants were provided financial compensation for their time of involvement in the study. Each interview lasted approximately 30–45 min. The study received approval from the University of Ibadan/University College Hospital Research Ethics Committee (IRB Research Number: NHREC/05/01/2008a).

### 2.4. Development of Interview Guide

Key informant interviews were conducted among OLHIV and HCPs using a structured guide developed based on the Consolidated Framework for Implementation Research (CFIR) focusing on its five domains: intervention characteristics, outer setting, inner setting, characteristics of individuals, and process. CFIR 1 provides a well‐stablished framework for identifying multilevel determinants of implementation and has been widely applied in studies of healthcare service integration. The interview guide had three sections: (1) existing structures/systems in the clinics; (2) factors that could affect integration of the intervention model; and (3) perceived feasibility and integration process of the SCT model. Questions were aligned with the five CFIR 1 domains. Section 1 addressed Domain 1 in CFIR 1 (inner settings), Section 2 focused on Domain 2 (outer setting), and Section 3 explored Domains 3 (individual characteristics), 4 (process), and 5 (intervention characteristics). This mapping ensured comprehensive coverage of relevant CFIR 1 constructs across all interview sections (Table 2). During the interview, the proposed intervention process was described for the purpose of feedback with the aim of assessing its feasibility and how best it fit or can be made fit into the current existing HIV services before pilot testing.

**TABLE 2:** Interview guide development framework.

CFIR 1 domain	Operationalization in the project
Inner setting	Contextual features of the HIV clinic (administrative structure and processes) shaping implementation
Outer setting	Factors outside of the clinic that influence patient care and intervention implementation.
Individual characteristics	Attributes of older adults living with HIV and health‐care providers delivering the intervention.
Process	Actions that patients and healthcare providers take to implement the stepped‐care task‐sharing intervention.
Intervention characteristics	Core components of a program to provide depression care for older adults living with HIV.

### 2.5. Data Collection

The interview team consisted of an interviewer and a note‐taker. The interviewer followed the structured interview guide, and the note‐taker simultaneously took notes in a structured format. The interviews were conducted by OE (MBBS, MSc), a psychiatrist, and FA (Mph), a public health researcher; both are women with formal training in qualitative methods and experience conducting in‐depth interviews in clinical and community settings. Interviews were conducted and audio‐recorded in English and Yoruba at the HIV clinic of the IDI, Ibadan. Participants were recruited via purposive sampling and were approached face‐to‐face to participate. The note‐taker was permitted to ask questions during the interview to obtain clarification on a concept or statement. Key statements from interviews completed in Yoruba were translated and documented in English by bilingual team members. Interviews lasted approximately 30–60 min. Upon completion of the interviews, data were securely stored on password‐protected, encrypted devices. Data were only accessible to authorized personnel via multifactor authentication.

### 2.6. Data Analysis

The qualitative team consisted of researchers with diverse expertise in both qualitative methods and implementation science (LB, BO, OE, LK, and AF). Two investigators reviewed detailed interview data with HCPs and OLHIV. Using Hamilton’s rapid qualitative analysis approach [20, 21], interview summaries were generated directly from the audio recordings, with key quotations incorporated into the summaries, eliminating the need for full transcriptions and facilitating rapid analysis [22]. One interview was jointly reviewed to refine the initial CFIR‐informed codebook and ensure consistency. Each analyst then independently summarized and categorized two additional interviews, cross‐checked their categorization, and resolved through discussion, leading to iterative refinement of the CFIR‐informed codebook. Once consensus was reached, the remaining interviews were divided between the two analysts and summarized within a structured CFIR‐based matrix to facilitate comparison across participants and identify key themes. Analysis combined deductive (CFIR‐informed) and inductive (emergent) coding to capture both expected and new implementation factors.

Coding was conducted by two team members (OE and AF) with backgrounds in psychiatry and public health respectively, who were not affiliated with participant organizations, involved in clinical care of PLWH, or living with HIV. We recognized that our professional backgrounds could influence interpretation of participant’s accounts. To enhance reflexivity, coding decisions and emergent themes were discussed iteratively within the research team, until consensus was reached. Interviews continued until coding saturation was achieved [23], with thematic saturation reached by the 10th patient interview and the eighth HCP interview. All authors reviewed and confirmed the final findings. Transcripts were not shared or returned to participants; however, analytic discussions among the research team enhanced credibility.

## 3. Results

### 3.1. Participant Demographics

In total, 11 HCPs: 6 nurses, 2 counselors, 1 record officer, and 2 doctors, were interviewed from the HIV clinic. The mean age of HCPs and OLHIV was 49·1 years (± 10·3) and 56·8 years (± 5·9), respectively (Table 2). Interviews were completed between February 8^th^, 2025, and February 20^th^, 2025. In Table 2, age ranges are presented, rather than exact ages to protect confidentiality. In addition, 11 OLHIV were interviewed, including 3 (27.3%) men and 8 (72.7%) women (Table 3). Median duration since HIV diagnosis 5 years was 19 years. The mean age of HCPs and OLHIV was 49·1 years (± 10·3) and 56·8 years (± 5·9), respectively (Table 2). Interviews were completed between February 8^th^, 2025, and February 20^th^, 2025. In Table 2, age ranges are presented, rather than exact ages to protect confidentiality.

**TABLE 3:** Demographic characteristics of the study participants.

OLHIV			Healthcare providers (HCPs)						
ID	Age (56.8 ± 5·9)	Sex	Level of education	ID	Age (49·1 ± 10·3)	Sex	Level of education	Cadre	Occupation
P1	45–54	Male	Tertiary	P1	45–54	Female	BSc	Public health nurse	Nurse
P2	55–60	Female	No formal Education	P2	55–60	Female	BNSc	Nurse/midwife	Nurse
P3	55–60	Female	No formal Education	P3	55–60	Female	BNSc	Public health nurse	Nurse
P4	65–70	Female	Tertiary	P4	55–60	Male	MSc	Counseling psychologist	Counselor
P5	45–54	Male	Tertiary	P5	45–54	Female	BSc	Health record	Record officer
P6	45–54	Female	Tertiary	P6	45–54	Female	BSc	Nurse	Nurse
P7	61–66	Female	No formal education	P7	25–30	Female	MBBS	Medical Officer	Physician
P8	45–54	Male	No formal Education	P8	35–44	Female	MBBS	Medical Officer	Physician
P9	45–54	Female	Tertiary	P9	45–54	Female	BNSc	Nurse	Nurse
P10	61–66	Female	Tertiary	P10	55–60	Female	MSc	Medical social worker	Social worker
P11	45–54	Female	No formal Education	P11	45–54	Female	BNSc	Nurse	Nurse

*Note:* BSc, Bachelor of Science; BNSc, Bachelor of Nursing Science; MSc, Master of Science; MBBS, Bachelor of Medicine, Bachelor of Surgery; OLHIV, older adults living with HIV.

### 3.2. Main Findings: Qualitative Themes

The rapid qualitative analysis revealed 11 key themes across the three primary sections from the interview guide mapped to CFIR domain, including Domain 1 (inner setting comprised 3 themes which explored existing psychological health services in HIV care, organizational structures, and infrastructural barriers), Domain 2 (outer setting, 2 themes related to patient needs and resources and stigma), Domain 3 (individual characteristics, 3 themes captured lack of readiness to implement stepped care, limited provider competence, and scheduling of appointments), Domain 4 (process, 2 number of themes relating to sustaining intervention delivery and access‐related barriers), and Domain 5 (intervention characteristics which described need for contextual adaptation) (Figure 1 and Appendix 1 (Table A1)). Each theme is presented below with illustrative key quotations from OLHIV and HCPs, as relevant.

**FIGURE 1:**
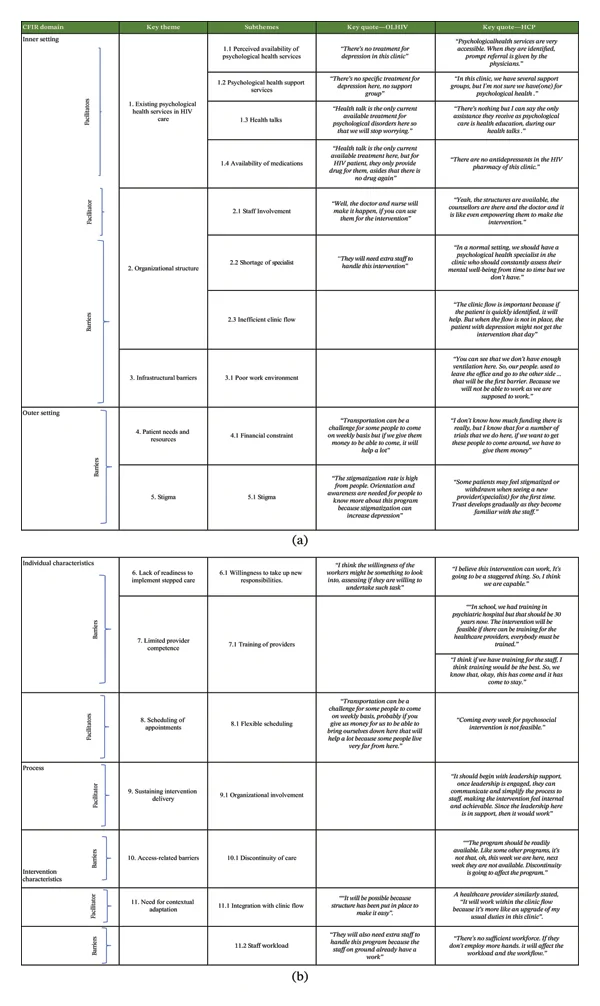
(a) Codebook table showing the themes and key quotes from participants. (b) Codebook table showing the themes and key quotes from participants.

### 3.3. Domain 1: Inner Setting

The inner setting describes the organizational characteristics of HIV clinics that influence the implementation of the SCT intervention. Within this domain, participants identified both facilitators and barriers to implementation relating to existing mental health resources and organizational structures with key themes relating to routine health talks (i.e., educational seminars offered to patients), organizational collaboration, staffing and resource limitations, and clinic infrastructure. Given the importance of health talks, they serve as a medium to sensitize and educate patients in a group setting about key health issues affecting their health and well‐being. Also, within the inner setting, staff involvement in the implementation process was identified as a facilitator. However, staffing shortages, inefficient clinic flows, and inadequate infrastructures were cited as barriers (Table 4).

**TABLE 4:** Recommendations for contextual adaptation of SCT intervention.

Recommendations	Key quotes—OLHIV	Key quotes—HCP
Capacity building		“*Training will empower the counselors. And it’s more like, upgrading yourself as a counselor. It could, now empower the counselors and further improve their work, and make depression easy to quickly detect.*
Availability of medications	*“It will work, for example, when they are thinking they will send to the doctor for counseling, and give them treatment like drug, so it’s going to work”.*	*There should be availability of drugs. The drugs we are supposed to prescribe for the patients to buy should be available in the clinic to avoid discontinuity*
Use of routine health talk	*“If there’s awareness and orientation for the program, it will be better”*	*“We already have health talks every morning, which can easily incorporate this intervention. Addressing them within the general group ensures everyone’s needs are met, followed by specific individual referral and follow-ups for depression. The existing morning health talks can be used for this purpose.”*
Flexible scheduling	*If some can’t come because of transport, you can bring the intervention down to every health centers.*	*“That might be a tough one. Making them come for the weekly assessments. You can batch it into sessions. So those that are not able to meet up with the first physical session can have theirs at the second or third drug pick up and make use of phone calls for the assessment and psychosocial intervention like we do in our enhanced adherence counseling.”* *“Instead of coming every week, you can do the psychological intervention on phone. Because if you tell them to come in 2 weeks, are you going to provide for their transport?”*
Monitoring and evaluation		*“Monitoring and evaluation like, okay, are we seeing improvements? Is it significant? Is it worth the extra investment? So, we have to be able to assess it after a while and compare and see if the intervention is working out”*

#### 3.3.1. Theme 1: Existing Psychological Health Services in HIV Care

This theme describes participants’ perceptions of the psychological health services currently available within the HIV clinic.

##### 3.3.1.1. Subtheme 1.1: Perceived Availability of Psychological Health Services

Participants expressed differing views regarding the availability of psychological health services within the clinic. While some HCPs reported that patients with psychological needs were promptly identified and referred for appropriate care, most HCPs and OLHIV perceived that no dedicated psychological or depression services were available within the clinic.

“Psychological health services are very accessible. When they are identified, prompt referral is given by the physicians.” (HCP, Nurse)

“There’s no treatment for depression in this clinic.” (OLHIV, Female)

##### 3.3.1.2. Subtheme 1.2: Psychological Health Support Services

Participants also reported limited availability of psychological support services. Although several patient support groups existed within the clinic, participants were unaware of any specifically designed to address psychological health or depression.

“In this clinic, we have several support groups, but I′m not sure we have [one] for psychological health.” (HCP, Nurse)

“There’s no specific treatment for depression here, no support group.” (OLHIV, Female)

##### 3.3.1.3. Subtheme 1.3: Health Talks

Although formal psychological health support was reported to be unavailable, participants identified routine health talks as the only existing form of support that resembled psychosocial support currently within the clinic. However, both HCPs and OLHIV noted that these sessions focused on general health education and encouragement rather than specialized mental healthcare.

“There’s nothing, but I can say the only assistance they receive as psychological care is health education during our health talks.” (HCP, Nurse)

“Health talk is the only current available treatment for psychological disorders here so that we will stop worrying.” (OLHIV, Female)

##### 3.3.1.4. Subtheme 1.4: Availability of Medications

Participants mentioned the need for the availability of medications that could facilitate the implementation of the intervention and the need for provision of free antidepressants.

A nurse mentioned that:

“There are no antidepressants in the HIV pharmacy of this clinic.” (HCP, Nurse)

“Health talk is the only current available treatment here, but for HIV patient, they only provide drug for them, asides that there is no drug again.” (OLHIV, Female)

#### 3.3.2. Theme 2: Organizational Structure

##### 3.3.2.1. Subtheme 2.1: Staff Involvement (Facilitator)

Participants identified the existing multidisciplinary workforce as an important facilitator for integrating depression care into routine HIV services. Physicians, nurses, and counselors were perceived to have the capacity to support depression screening and management if provided with appropriate training.

“Well, the doctor and nurse will make it happen, if you can use them for the intervention.” (OLHIV, Male)

“Yeah, the structures are available, the counselors are there and the doctor and it is like even empowering them to make the intervention.” (HCP, Counselor)

##### 3.3.2.2. Subtheme 2.2: Shortage of Specialist (Barrier)

Despite recognizing the capacity of existing staff, participants identified shortages of healthcare personnel as a barrier to implementation. The absence of dedicated mental health specialists and concerns about increased workload were perceived as limiting the clinic’s ability to provide ongoing depression care.

“In a normal setting, we should have a psychological health specialist in the clinic who should constantly assess their mental well‐being from time to time but we don’t have.” (HCP, Counselor)

“They will need extra staff to handle this intervention since it’s going to be a continuous process.” (OLHIV, Female)

##### 3.3.2.3. Subtheme 2.3: Inefficient Clinic Flow (Barrier)

Participants also described inefficient clinic flow as a barrier to timely depression identification and management. They noted that delays in patient movement through the clinic could reduce opportunities to screen and provide appropriate intervention during the same visit.

“The clinic flow is important because if the patient is quickly identified, it will help. But when the flow is not in place, the patient with depression might not get the intervention that day.” (HCP, Medical Officer)

#### 3.3.3. Theme 3: Infrastructural Barriers

##### 3.3.3.1. Subtheme 3.1: Poor Work Environment (Barrier)

Participants described poor working conditions, inadequate ventilation, and clinic environments as challenges:

“You can see that we don’t have enough ventilation here. So, our people. used to leave the office and go to the other side … that will be the first barrier. Because we will not be able to work as we are supposed to work.” (HCP, Record officer)”

### 3.4. Domain 2: Outer Setting

The outer setting domain examines how external factors outside the HIV clinic, including patient needs, community contexts, and health systems, facilitate or hinder implementation of the intervention. Findings were organized into 2 themes: Theme 1: financial constraints and Theme 2: stigma, which were identified as barriers to incorporating the SCT into routine care while facilitators include routine health talk sessions, availability of health personnel, nurses’ training, access to medical doctors for referrals, and dedicated counselors (Table 3).

#### 3.4.1. Theme 4: Patient Needs and Resources

##### 3.4.1.1. Subtheme 4.1: Financial Constraint (Barrier)

Participants highlighted the need for financial resources in terms of monetary support to be available for patients as this serves as a tool for effective care.

“Transportation can be a challenge for some people to come on weekly basis but if we give them money to be able to come, it will help a lot” (OLHIV, Female)

Another said: “I don’t know how much funding there is really, but I know that for a number of trials that we do here, if we want to get these people to come around, we have to give them money” (HCP, Medical Officer).

#### 3.4.2. Theme 5: Stigma

##### 3.4.2.1. Subtheme 5.1: Stigma

Participants reported stigma as a barrier and considered building trust over time as essential for effective intervention.

“Some patients may feel stigmatized or withdrawn when seeing a new provider(specialist) for the first time. Trust develops gradually as they become familiar with the staff.” (HCP, Nurse)

“On the part of the OLHIV they mentioned that”*:*

“The stigmatization rate is high from people. Orientation and awareness are needed for people to know more about this program because stigmatization can increase depression” (OLHIV, Female)

### 3.5. Domain 3: Individual Characteristics

Individual characteristics explored how HCPs’ knowledge, beliefs, skills, and motivation influenced implementation. Findings were organized into three subthemes to illustrate how individual‐level factors shaped readiness and capacity to deliver the stepped‐care intervention.

At the individual level, HCPs expressed willingness to incorporate psychological health services but identified inadequate training as a barrier (Table 3). However, providers reported they are willing to take responsibility if given proper training and supervision.

#### 3.5.1. Theme 6: Lack of Readiness to Implement Stepped Care (Barrier)

##### 3.5.1.1. Subtheme 6.1: Unwillingness of HCPs to Take Up New Responsibilities

The importance of the willingness of healthcare workers to adopt the intervention was mentioned, and this was reported as important for the success of the intervention:

“I think the willingness of the workers might be something to look into, assessing if they are willing to undertake such task” (OLHIV, Female)

Another stated that “I believe this intervention can work, It’s going to be a staggered thing. So, I think we are capable.” (HCP, Nurse)

#### 3.5.2. Theme 7: Limited Provider Competence

##### 3.5.2.1. Subtheme 7.1: Training of Providers (Facilitator)

HCPs highlighted the need for adequate training that could help improve their skill in providing psychological intervention for depression.

“In school, we had training in psychiatric hospital but that should be 30 years now. The intervention will be feasible if there can be training for the healthcare providers, everybody must be trained.” Another reinforced the need for it saying:

“I think if we have training for the staff, I think training would be the best. So, we know that, okay, this has come and it has come to stay” (HCP, Nurse)

#### 3.5.3. Theme 8: Scheduling of Appointments

##### 3.5.3.1. Subtheme 8.1: Flexible Scheduling (Facilitator)

Participants described the need to reach patients physically. However, weekly visits for psychosocial intervention were presented as not feasible. It was proposed that intervention could be scheduled into their clinic visit days or when they come for drug pick‐up noting that:

“Transportation can be a challenge for some people to come on weekly basis, probably if you give us money for us to be able to bring ourselves down here that will help a lot because some people live very far from here.” (OLHIV, Female)

“Coming every week for psychosocial intervention is not feasible” (HCP, Medical Officer)

### 3.6. Domain 4: Process

Domain 4 describes how the intervention could be integrated into existing HIV clinic settings, including the planning, engagement, execution, and coordination activities that support implementation. Within the process domain, providers raised concerns about sustaining interventions over time and mentioned discontinuity of care as a challenge. Strong leadership support and engagement, however, were mentioned as key facilitators.

#### 3.6.1. Theme 9: Sustaining Intervention Delivery

##### 3.6.1.1. Subtheme 9.1: Organizational Involvement in Sustaining Intervention Delivery (Facilitator)

HCPs identified leadership engagement as a key facilitator of sustained intervention delivery. They noted that support from organizational leaders would promote staff buy‐in, facilitate communication, and integrate the intervention into routine practice.

“It should begin with leadership support, once leadership is engaged, they can communicate and simplify the process to staff, making the intervention feel internal and achievable. Since the leadership here is in support, then it would work” (HCP, Medical Officer))

#### 3.6.2. Theme 10: Access‐Related Barriers

##### 3.6.2.1. Subtheme 10.1: Discontinuity of Care (Barrier)

HCPs pointed out the need for continuity and sustenance of the intervention for it to be considered successful:

HCP mentioned that: “The program should be readily available. Like some other programs, it’s not that, oh, this week we are here, next week they are not available. Discontinuity is going to affect the program” (HCP, Nurse)

### 3.7. Domain 5: Intervention Characteristics

This domain explores how the design and characteristics of the stepped‐care intervention, including its adaptability and perceived feasibility, may facilitate or hinder its integration into routine HIV care. Participants described the need for intervention to be contextually adapted for proper integration into existing workflow. Facilitator subthemes included integration into clinic flow and staff workload (Table 3).

#### 3.7.1. Theme 11: Need for Contextual Adaptation

##### 3.7.1.1. Subtheme 11.1: Integration With Clinic Flow (Facilitator)

HCPs believed the intervention could be integrated into existing clinic workflows, describing it as an extension of their routine responsibilities. Similarly, OLHIV perceived that the intervention could be incorporated into current service delivery because of existing clinic structures.

As one participant explained, “It will be possible, because structure has been put in place to make it easy” (OLHIV, Female). A healthcare provider similarly stated, “It will work within the clinic flow because it’s more like an upgrade of my usual duties in this clinic” (HCP, Nurse).

##### 3.7.1.2. Subtheme 11.2: Staff Workload (Barrier)

HCPs expressed concerns that delivering the intervention could increase their workload and disrupt routine clinical activities. They suggested the need for additional health personnel to ensure the intervention could be implemented effectively without overburdening existing staff.

One of the participants said: “They will also need extra staff to handle this program because the staff on ground already have a work” (OLHIV, Female)

“There’s no sufficient workforce. If they don’t employ more hands. It will affect the workload and the workflow.” (HCP, Record officer).

### 3.8. Recommendations for the Contextual Adaptation of the SCT Model

Using the CFIR domains, participants recommended factors that would support successful integration of depression care into the existing HIV clinic. They highlighted the need for capacity building, availability of medications, use of routine health talks, flexible scheduling, and ensuring monitoring and evaluation (see Table 4).

## 4. Discussion

Guided by CFIR and using qualitative rapid analysis, this preimplementation study examined the feasibility of integrating and contextual fit of the SCT model for treatment of depression into existing HIV services among OLHIV in Ibadan. Integration was considered feasible due to staff willingness and existing structures. However, contextual adaptation such as aligning interventional and clinic workflows and clarifying task‐sharing roles were required to support practical implementation. Key barriers included stigma, workload, provider‐related discontinuity of care, and limited funding while organizational readiness and user acceptability were identified as important facilitators.

Currently, integration of psychological health into HIV services remains limited in this setting, reflecting persistent structural and perceptual gaps. Although providers described referrals to external psychiatric clinics as available psychological health services, patients did not view this as part of their routine HIV care. This disconnect highlights a weak and poorly integrated referral pathway, a challenge also reported in Ghana and Kenya, where referral‐based models were limited by poor patient awareness [24, 25]. These findings suggest that referral‐only approaches may be insufficient to meet the psychological health needs of PLWH and underscore the need for services embedded within HIV clinics.

Existing clinic‐based communication and health promotion platforms such as “health talks” emerged as an important opportunity for integration. Although health talk sessions were largely informational and not specifically designed to address psychological health disorders, their prominence within routine clinic positions them as feasible entry points for brief psychological health education, screening, and referral to receive treatment for depression within the HIV clinic. Similar approaches in Uganda and Malawi demonstrated that embedding low‐intensity psychological health activities into existing clinic routines enhanced feasibility and patient participation [26, 27], supporting the potential adaptability of this strategy in Nigerian HIV clinics.

Within the inner setting, staff involvement and organizational readiness were key facilitators for integrating the SCT model into HIV services. Consistent with findings from sub‐Saharan Africa, active involvement of leadership and frontline workers promotes ownership and long‐term sustainability of interventions [28, 29]. In contexts of limited specialist availability, the SCT model offers a practical approach by redistributing care responsibilities to trained nonspecialist providers [30]. Evidence from Cameroon and other sub‐Saharan African countries demonstrates that task sharing can build nonspecialist capacity to identify and manage common psychological disorders, further supporting the relevance of this approach [29, 31, 32].

At the outer setting level, stigma and financial constraints emerged as barriers influencing implementation. Facilitators include routine health talk sessions, availability of health personnel, nurses’ training, access to medical doctors for referrals, and dedicated counselors. Limited awareness and knowledge about depression continue to shape community perceptions and contribute to stigma, which negatively affects service uptake and engagement [33]. This finding aligns with extensive evidence from HIV care settings across sub‐Saharan Africa, where stigma remains a major impediment to integrated psychological healthcare delivery and sustainability [27, 33–35].

Participants also identified financial constraints as a potential barrier to sustained engagement with the intervention, particularly the costs of repeated clinic visits for psychosocial interventions and antidepressant treatment. Similar financial barriers have been reported among PLWH in Uganda, where out‐of‐pocket expenses reduce engagement with care [36]. Integrating depression care into routine HIV visits may help minimize these costs and improve sustained participation. Addressing these contextual barriers will be essential to achieving meaningful integration.

Individual‐level factors also influenced feasibility. Providers expressed openness to incorporating psychological health services into routine HIV services but identified inadequate training and skills as key limitations. Importantly, providers indicated willingness to assume these roles if supported through appropriate training and supervision. This is consistent with prior studies emphasizing the role of skill‐building, provider confidence, and competence as factors that could significantly enhance implementation success [37, 38]. From the patient’s perspective, flexible appointment scheduling and financial support were identified as important to sustain engagements in care, strategies that have been known to improve retention on other HIV and psychological health programs in low‐resource settings [26, 27].

Concerns regarding sustainability and continuity of care were raised within the process domain. Nonetheless, strong leadership engagement emerged as a key facilitator, reinforcing evidence that leadership commitment is essential for successful implementation and long‐term sustainability of integrated care models [29, 39].

Regarding intervention characteristics, providers expressed concerns about potential increase in workload with the intervention. However, both patients and providers perceived the SCT model could fit well within HIV care workflows if appropriately adapted. Suggested strategies for enhancing feasibility included training and supervision of nonspecialist staff by the specialists, flexible scheduling of appointments, ensuring medication availability, and leveraging existing clinic infrastructures. Collectively, these findings suggest that integration of depression care into HIV programs in Nigeria is feasible but is contingent on addressing key implementation barriers while building on existing facilitators. Next steps include adapting the model using these findings, conducting pilot testing, and scaling the intervention across diverse HIV care settings.

## 5. Conclusion

This preimplementation assessment shows that the SCT model for depression is acceptable and potentially feasible for OLHIV but requires deliberate contextual adaptation to fit existing clinic structures and patient needs. Provider‐ and patient‐informed refinements highlight the importance of organizational support, streamlined workflows, provider capacity building, and flexible scheduling for effective delivery. Incorporating these adaptations before pilot testing lays the foundation for scalable integration of psychological healthcare within routine HIV services in resource‐constrained settings.

### 5.1. Limitations

The predominance of female participants may have influenced the findings, particularly on help‐seeking behaviors and the acceptability of task‐sharing, given that women are generally more likely to engage with health services in this setting. This may limit generalizability of findings to clinics in which woman are the majority of patients.

## Funding

Research reported in this manuscript was supported by the Royal Society of Tropical Medicine and Hygiene (RSTMH) under Award Number nihr24156 and the Fogarty International Center of the National Institutes of Health under Award Number D43TW009608. Support institutes for this project include Office of the Director, National Institutes of Health (OD), Fogarty International Center (FIC), Office of Behavioral and Social Sciences Research (OBSSR), and National Institute of Mental Health (NIMH).

## Disclosure

The content is solely the responsibility of the authors and does not necessarily represent the official views of the RSTMH and National Institutes of Health.

## Conflicts of Interest

The authors declare no conflicts of interest.

## Data Availability

The data that support the findings of this study are available from the corresponding author upon reasonable request.

## Appendix 1

**TABLE A1:** Coding tree (CFIR‐informed + emergent themes).

CFIR domain	Theme	Subtheme	Codes
Inner setting	1. Existing HIV and mental health services	1.1 Perceived availability of psychological health services	Code: No depression treatment Code: Referral
		1.2 Psychological health support services	Code: Psychological health support services Code: Referral to care
		1.3 Health talks	Code: Only treatment is health talk
		1.4 Availability of medications	Code: No provision of medication
	2. Organizational structure	2.1 Staff involvement	Code: The doctor and nurse will help
		2.2 Shortage of specialist	Code: Specialist limitations
		2.3 Inefficient clinic flow	Code: Poor clinic flow
	3. Infrastructural barriers	3.1 Poor work environment	Code: Barriers in workspace

Outer setting	4. Patient needs and resources	4.1 Financial constraint	Code: Financial provision for patients
	5. Stigma	5.1 Stigma	Code: Wrong diagnosis of depression

Individual characteristics	6. Lack of readiness to implement care	6.1 Willingness to take up new responsibilities	Code: Openness to task sharing/leadership support
	7. Limited provider competence	7.1 Training of providers	Code: Healthcare providers’ capacity
	8. Scheduling of appointments	8.1 Flexible scheduling	Code: Incorporating screening into appointments

Implementation process	9. Sustaining intervention delivery	9.1 Organizational involvement	Code: Leadership support/staff involvement
	10. Access‐related barriers	10.1 Discontinuity of care	Code: Consistency with intervention

Intervention characteristics	11. Need for contextual adaptation	11.1 Integration with clinic flow	Code: Poor follow‐up
		11.2 Staff workload	Code: Overburdened staff

*Note:* This table presents the hierarchical coding structure developed using a deductive–inductive approach. Initial coding was guided by the Consolidated Framework for Implementation Research (CFIR) domains, with additional emergent themes incorporated iteratively during analysis.
